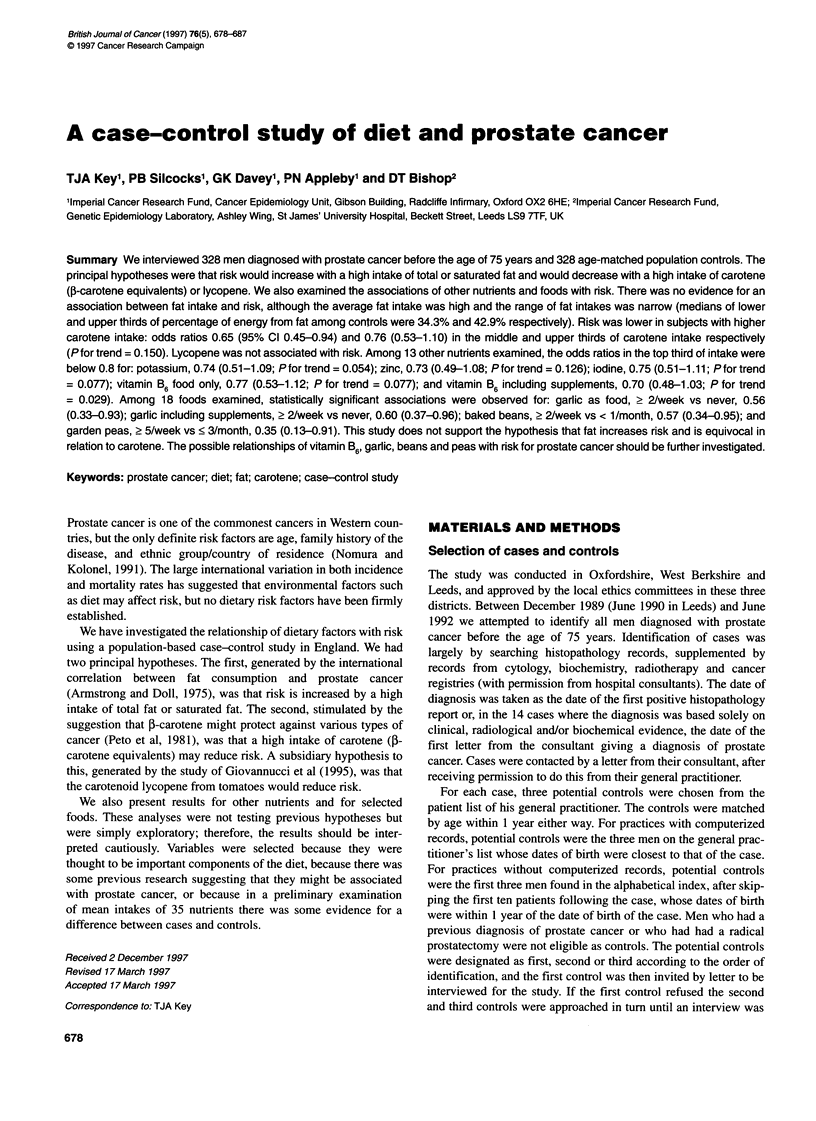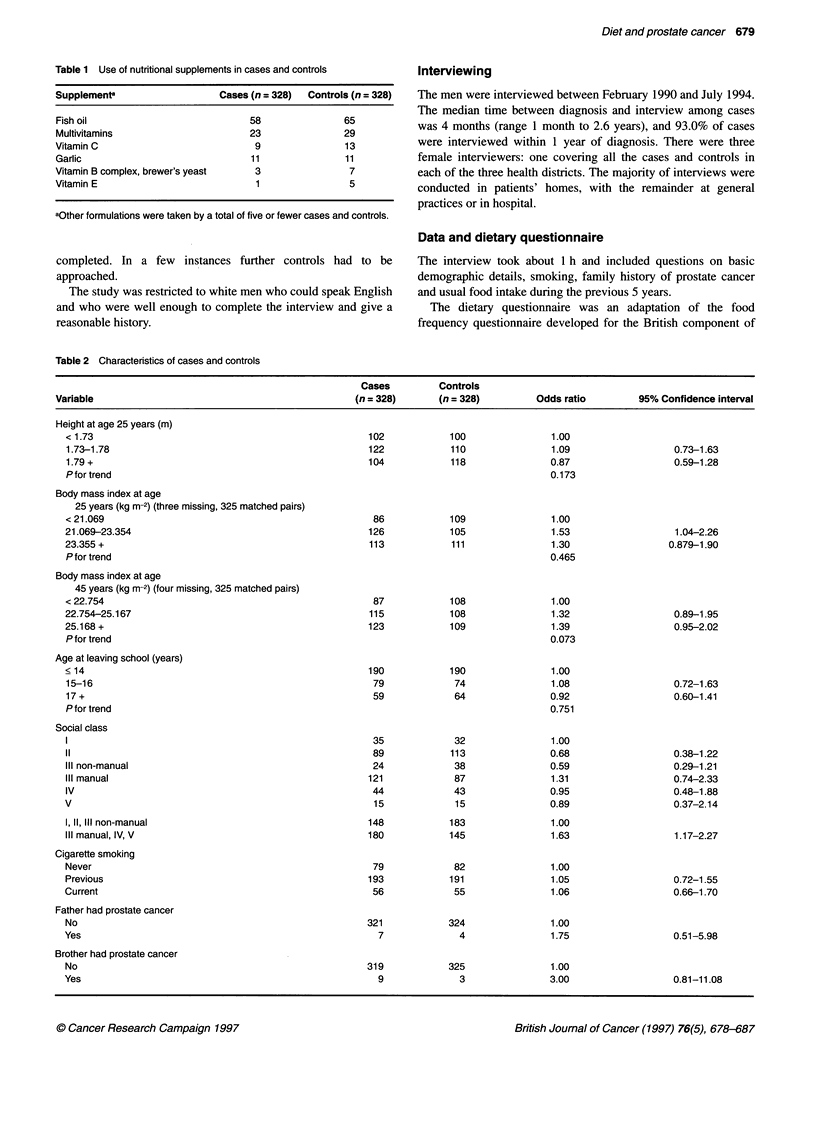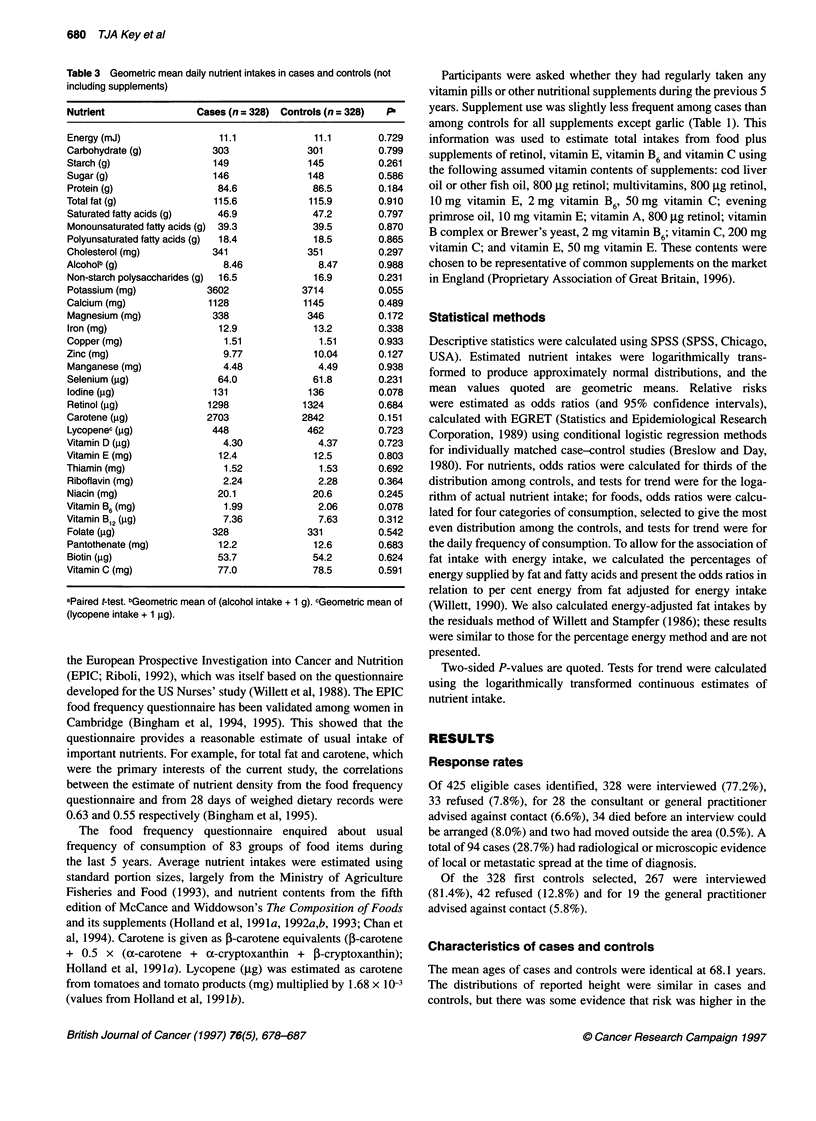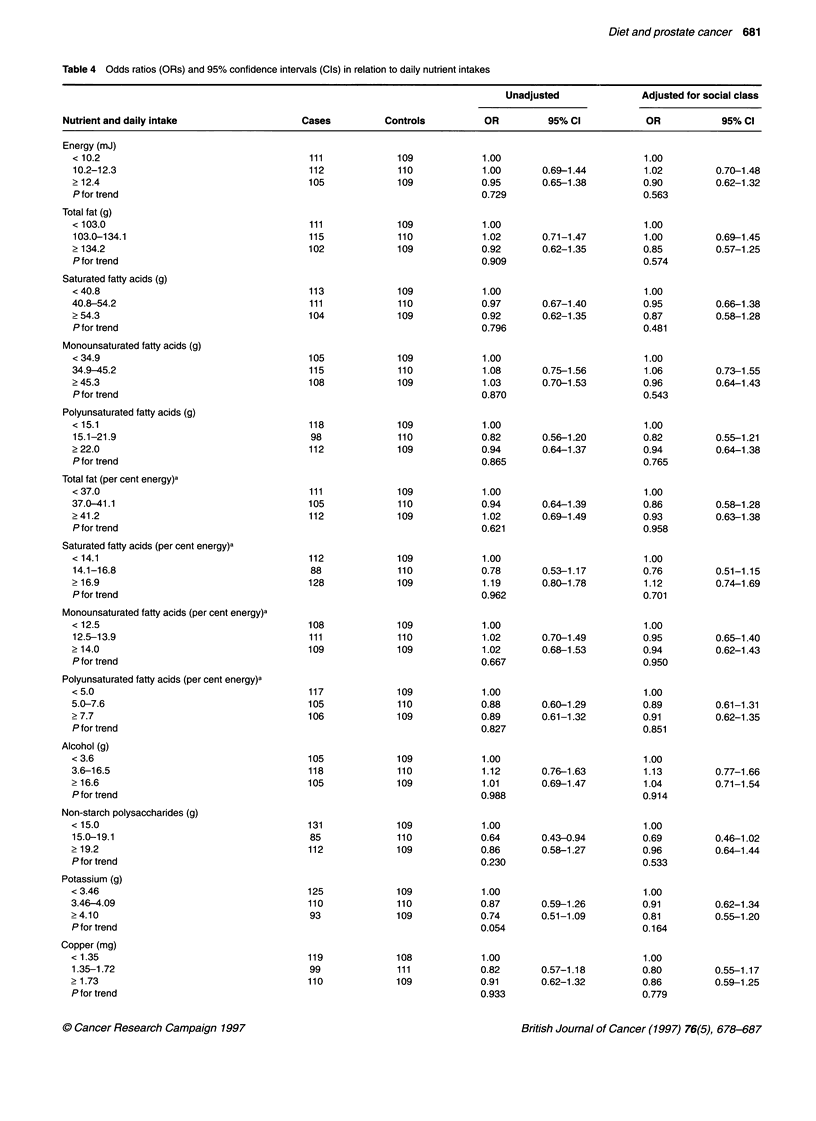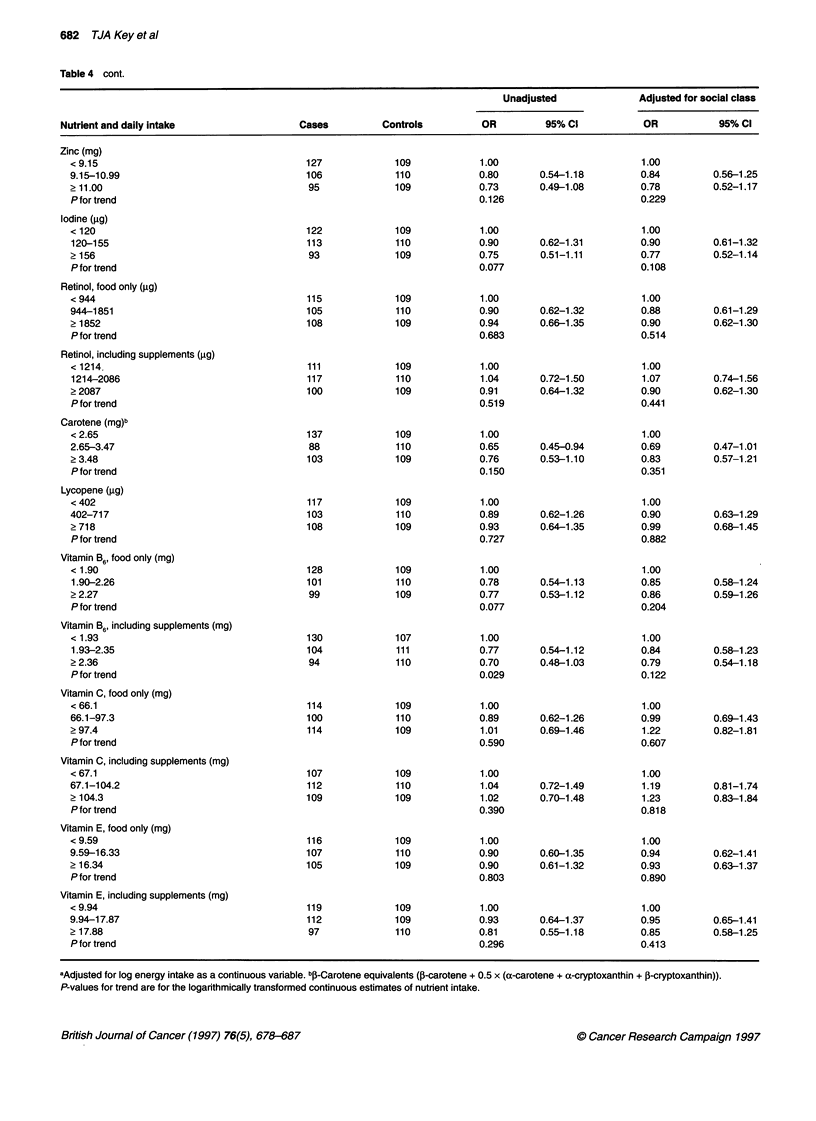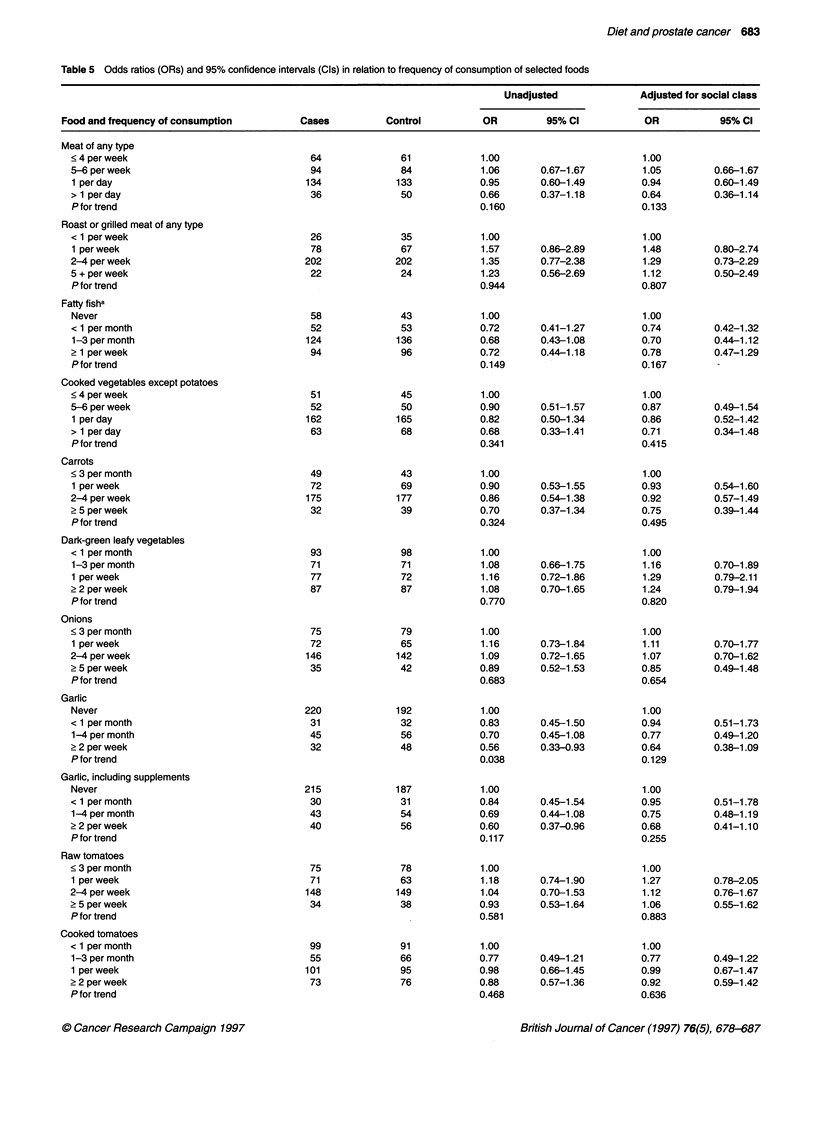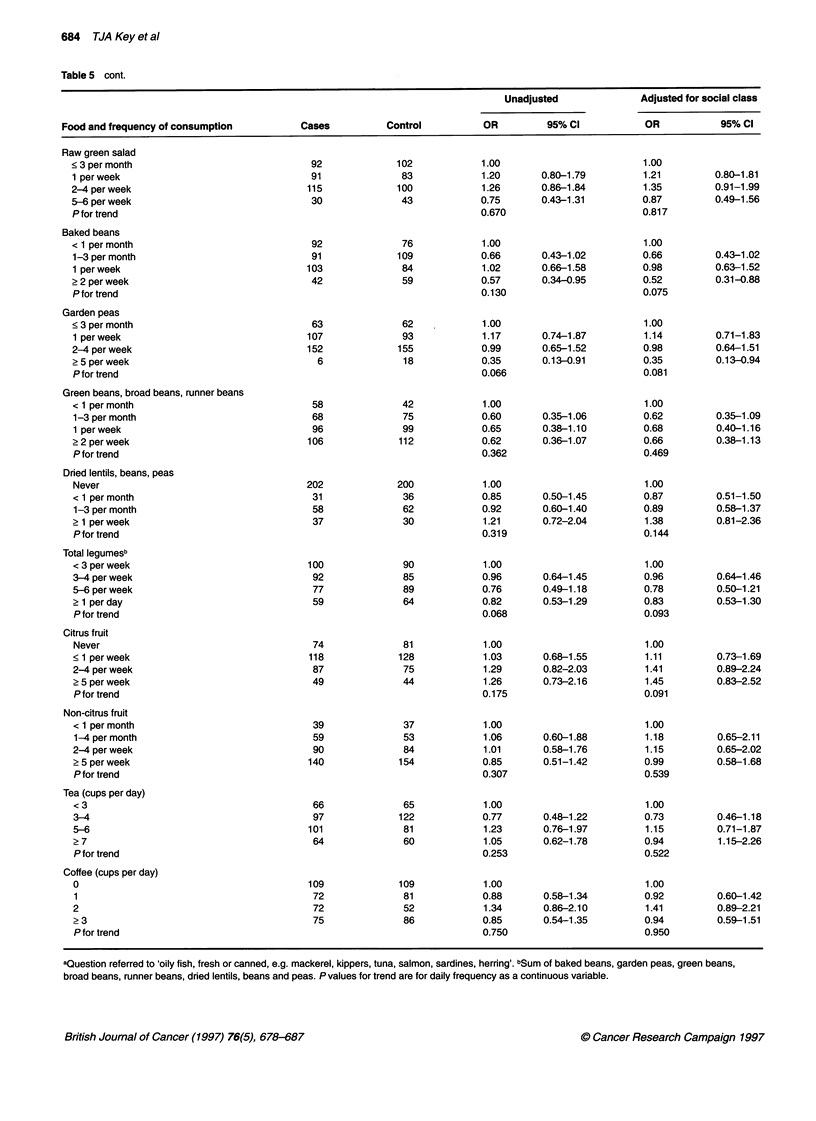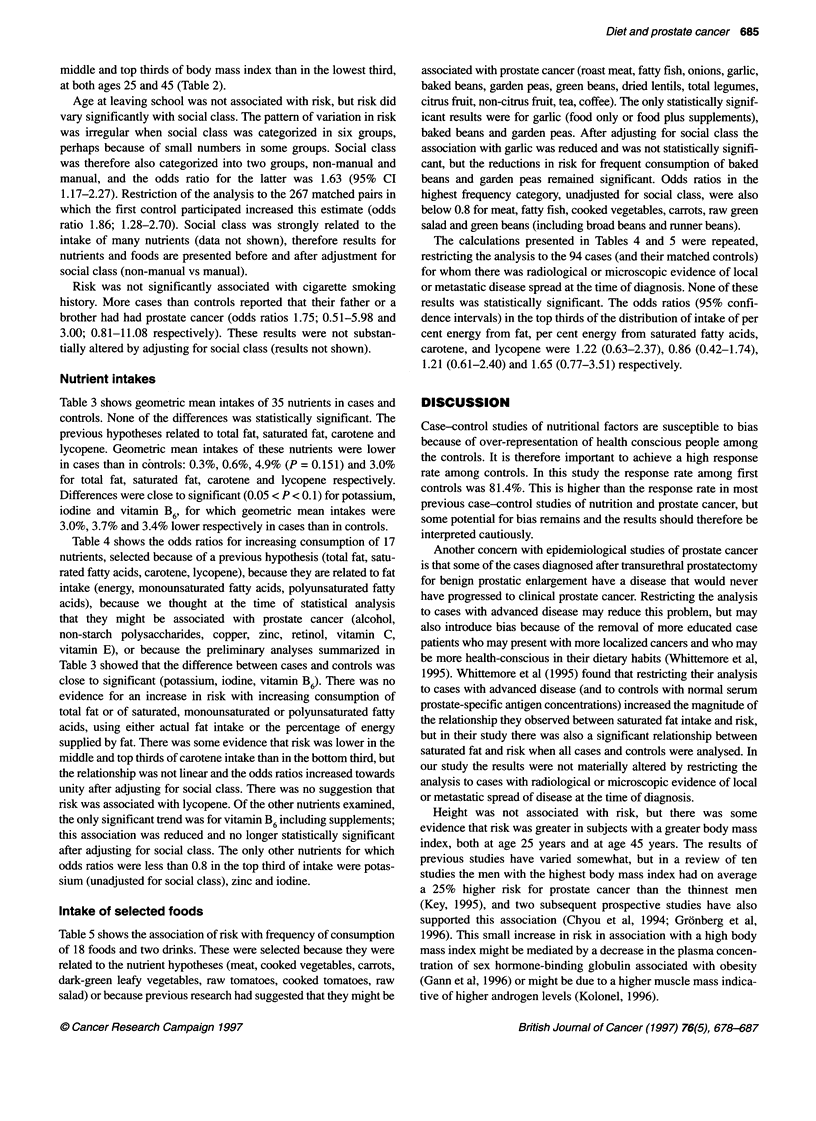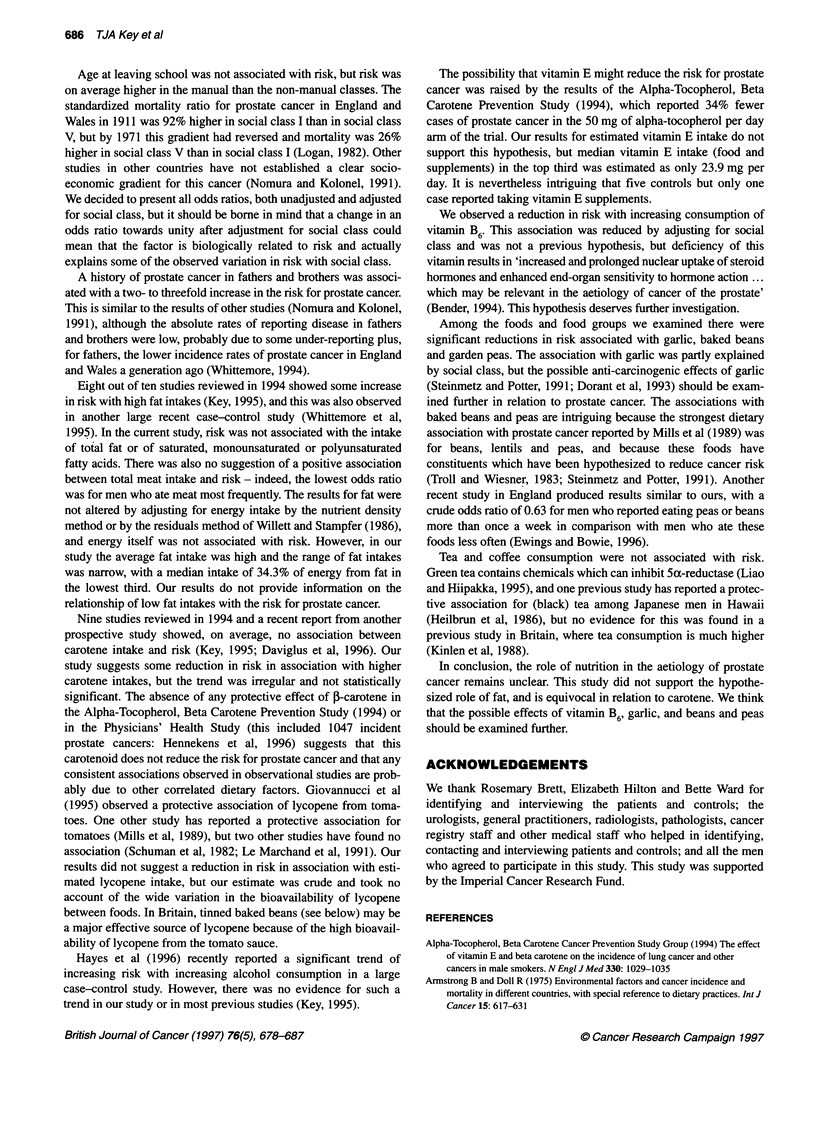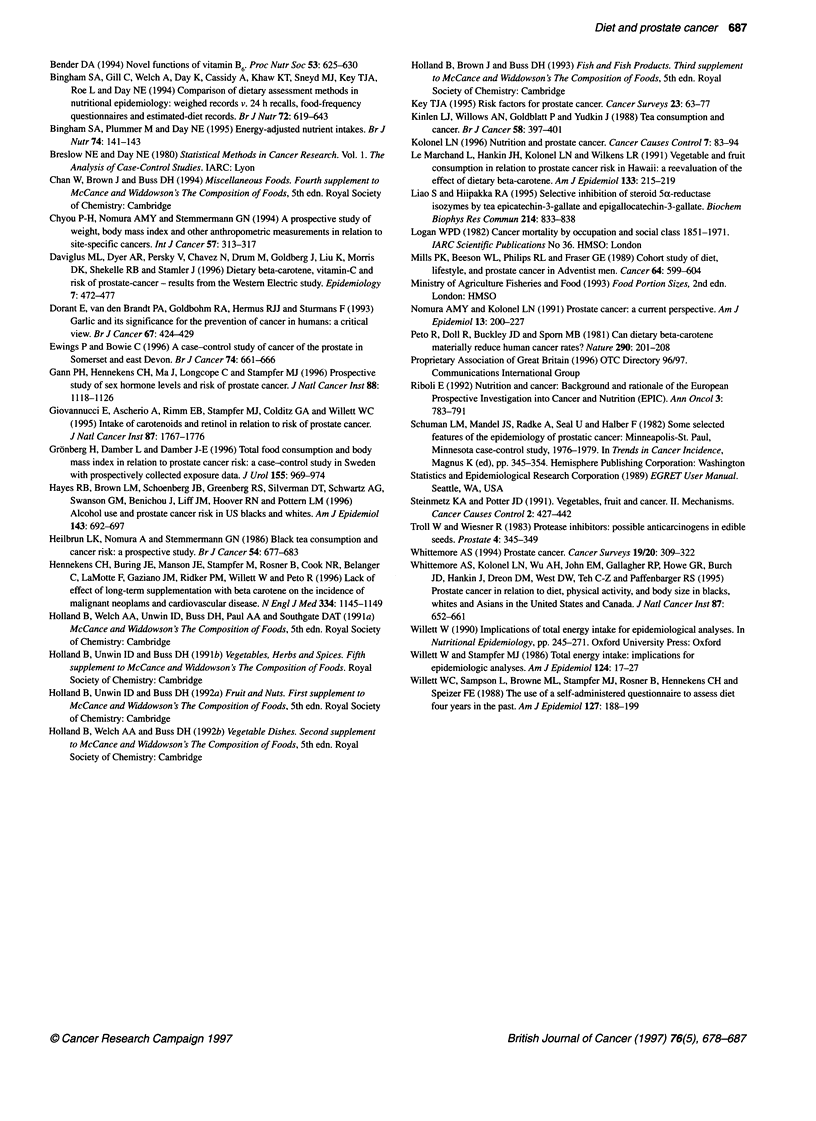# A case-control study of diet and prostate cancer.

**DOI:** 10.1038/bjc.1997.445

**Published:** 1997

**Authors:** T. J. Key, P. B. Silcocks, G. K. Davey, P. N. Appleby, D. T. Bishop

**Affiliations:** Imperial Cancer Research Fund, Cancer Epidemiology Unit, Radcliffe Infirmary, Oxford, UK.

## Abstract

We interviewed 328 men diagnosed with prostate cancer before the age of 75 years and 328 age-matched population controls. The principal hypotheses were that risk would increase with a high intake of total or saturated fat and would decrease with a high intake of carotene (beta-carotene equivalents) or lycopene. We also examined the associations of other nutrients and foods with risk. There was no evidence for an association between fat intake and risk, although the average fat intake was high and the range of fat intakes was narrow (medians of lower and upper thirds of percentage of energy from fat among controls were 34.3% and 42.9% respectively). Risk was lower in subjects with higher carotene intake: odds ratios 0.65 (95% CI 0.45-0.94) and 0.76 (0.53-1.10) in the middle and upper thirds of carotene intake respectively (P for trend = 0.150). Lycopene was not associated with risk. Among 13 other nutrients examined, the odds ratios in the top third of intake were below 0.8 for: potassium, 0.74 (0.51-1.09; P for trend = 0.054); zinc, 0.73 (0.49-1.08; P for trend = 0.126); iodine, 0.75 (0.51-1.11; P for trend = 0.077); vitamin B6 food only, 0.77 (0.53-1.12; P for trend = 0.077); and vitamin B6 including supplements, 0.70 (0.48-1.03; P for trend = 0.029). Among 18 foods examined, statistically significant associations were observed for: garlic as food, > or = 2/week vs never, 0.56 (0.33-0.93); garlic including supplements, > or = 2/week vs never, 0.60 (0.37-0.96); baked beans, > or = 2/week vs < 1/month, 0.57 (0.34-0.95); and garden peas, > or = 5/week vs < or = 3/month, 0.35 (0.13-0.91). This study does not support the hypothesis that fat increases risk and is equivocal in relation to carotene. The possible relationships of vitamin B6, garlic, beans and peas with risk for prostate cancer should be further investigated.


British Journal of Cancer (1997) 76(5), 678–687
© 1997 Cancer Research Campaign

### A case–control study of diet and prostate cancer

**TJA Key[1], PB Silcocks[1], GK Davey[1], PN Appleby[1] and DT Bishop[2]**

[1]Imperial Cancer Research Fund, Cancer Epidemiology Unit, Gibson Building, Radcliffe Infirmary, Oxford OX2 6HE; [2]Imperial Cancer Research Fund, Genetic Epidemiology Laboratory, Ashley Wing, St James' University Hospital, Beckett Street, Leeds LS9 7TF, UK

**Summary** We interviewed 328 men diagnosed with prostate cancer before the age of 75 years and 328 age-matched population controls. The principal hypotheses were that risk would increase with a high intake of total or saturated fat and would decrease with a high intake of carotene (β-carotene equivalents) or lycopene. We also examined the associations of other nutrients and foods with risk. There was no evidence for an association between fat intake and risk, although the average fat intake was high and the range of fat intakes was narrow (medians of lower and upper thirds of percentage of energy from fat among controls were 34.3% and 42.9% respectively). Risk was lower in subjects with higher carotene intake: odds ratios 0.65 (95% CI 0.45–0.94) and 0.76 (0.53–1.10) in the middle and upper thirds of carotene intake respectively (*P* for trend = 0.150). Lycopene was not associated with risk. Among 13 other nutrients examined, the odds ratios in the top third of intake were below 0.8 for: potassium, 0.74 (0.51–1.09; *P* for trend = 0.054); zinc, 0.73 (0.49–1.08; *P* for trend = 0.126); iodine, 0.75 (0.51–1.11; *P* for trend = 0.077); vitamin $B_6$ food only, 0.77 (0.53–1.12; *P* for trend = 0.077); and vitamin $B_6$ including supplements, 0.70 (0.48–1.03; *P* for trend = 0.029). Among 18 foods examined, statistically significant associations were observed for: garlic as food, ≥ 2/week vs never, 0.56 (0.33–0.93); garlic including supplements, ≥ 2/week vs never, 0.60 (0.37–0.96); baked beans, ≥ 2/week vs < 1/month, 0.57 (0.34–0.95); and garden peas, ≥ 5/week vs ≤ 3/month, 0.35 (0.13–0.91). This study does not support the hypothesis that fat increases risk and is equivocal in relation to carotene. The possible relationships of vitamin $B_6$, garlic, beans and peas with risk for prostate cancer should be further investigated.

**Keywords:** prostate cancer; diet; fat; carotene; case–control study

Prostate cancer is one of the commonest cancers in Western countries, but the only definite risk factors are age, family history of the disease, and ethnic group/country of residence (Nomura and Kolonel, 1991). The large international variation in both incidence and mortality rates has suggested that environmental factors such as diet may affect risk, but no dietary risk factors have been firmly established.

We have investigated the relationship of dietary factors with risk using a population-based case–control study in England. We had two principal hypotheses. The first, generated by the international correlation between fat consumption and prostate cancer (Armstrong and Doll, 1975), was that risk is increased by a high intake of total fat or saturated fat. The second, stimulated by the suggestion that β-carotene might protect against various types of cancer (Peto et al, 1981), was that a high intake of carotene (β-carotene equivalents) may reduce risk. A subsidiary hypothesis to this, generated by the study of Giovannucci et al (1995), was that the carotenoid lycopene from tomatoes would reduce risk.

We also present results for other nutrients and for selected foods. These analyses were not testing previous hypotheses but were simply exploratory; therefore, the results should be interpreted cautiously. Variables were selected because they were thought to be important components of the diet, because there was some previous research suggesting that they might be associated with prostate cancer, or because in a preliminary examination of mean intakes of 35 nutrients there was some evidence for a difference between cases and controls.

*Received 2 December 1997*
*Revised 17 March 1997*
*Accepted 17 March 1997*

*Correspondence to:* TJA Key

### MATERIALS AND METHODS

### Selection of cases and controls

The study was conducted in Oxfordshire, West Berkshire and Leeds, and approved by the local ethics committees in these three districts. Between December 1989 (June 1990 in Leeds) and June 1992 we attempted to identify all men diagnosed with prostate cancer before the age of 75 years. Identification of cases was largely by searching histopathology records, supplemented by records from cytology, biochemistry, radiotherapy and cancer registries (with permission from hospital consultants). The date of diagnosis was taken as the date of the first positive histopathology report or, in the 14 cases where the diagnosis was based solely on clinical, radiological and/or biochemical evidence, the date of the first letter from the consultant giving a diagnosis of prostate cancer. Cases were contacted by a letter from their consultant, after receiving permission to do this from their general practitioner.

For each case, three potential controls were chosen from the patient list of his general practitioner. The controls were matched by age within 1 year either way. For practices with computerized records, potential controls were the three men on the general practitioner's list whose dates of birth were closest to that of the case. For practices without computerized records, potential controls were the first three men found in the alphabetical index, after skipping the first ten patients following the case, whose dates of birth were within 1 year of the date of birth of the case. Men who had a previous diagnosis of prostate cancer or who had had a radical prostatectomy were not eligible as controls. The potential controls were designated as first, second or third according to the order of identification, and the first control was then invited by letter to be interviewed for the study. If the first control refused the second and third controls were approached in turn until an interview was

**Table 1** Use of nutritional supplements in cases and controls

| Supplement[a] | Cases ($n$ = 328) | Controls ($n$ = 328) |
|---|---|---|
| Fish oil | 58 | 65 |
| Multivitamins | 23 | 29 |
| Vitamin C | 9 | 13 |
| Garlic | 11 | 11 |
| Vitamin B complex, brewer's yeast | 3 | 7 |
| Vitamin E | 1 | 5 |

[a]Other formulations were taken by a total of five or fewer cases and controls.

completed. In a few instances further controls had to be approached.

The study was restricted to white men who could speak English and who were well enough to complete the interview and give a reasonable history.

### Interviewing

The men were interviewed between February 1990 and July 1994. The median time between diagnosis and interview among cases was 4 months (range 1 month to 2.6 years), and 93.0% of cases were interviewed within 1 year of diagnosis. There were three female interviewers: one covering all the cases and controls in each of the three health districts. The majority of interviews were conducted in patients' homes, with the remainder at general practices or in hospital.

### Data and dietary questionnaire

The interview took about 1 h and included questions on basic demographic details, smoking, family history of prostate cancer and usual food intake during the previous 5 years.

The dietary questionnaire was an adaptation of the food frequency questionnaire developed for the British component of

**Table 2** Characteristics of cases and controls

| Variable | Cases ($n$ = 328) | Controls ($n$ = 328) | Odds ratio | 95% Confidence interval |
|---|---|---|---|---|
| Height at age 25 years (m) | | | | |
| < 1.73 | 102 | 100 | 1.00 | |
| 1.73–1.78 | 122 | 110 | 1.09 | 0.73–1.63 |
| 1.79 + | 104 | 118 | 0.87 | 0.59–1.28 |
| *P* for trend | | | 0.173 | |
| Body mass index at age 25 years (kg m$^{-2}$) (three missing, 325 matched pairs) | | | | |
| < 21.069 | 86 | 109 | 1.00 | |
| 21.069–23.354 | 126 | 105 | 1.53 | 1.04–2.26 |
| 23.355 + | 113 | 111 | 1.30 | 0.879–1.90 |
| *P* for trend | | | 0.465 | |
| Body mass index at age 45 years (kg m$^{-2}$) (four missing, 325 matched pairs) | | | | |
| < 22.754 | 87 | 108 | 1.00 | |
| 22.754–25.167 | 115 | 108 | 1.32 | 0.89–1.95 |
| 25.168 + | 123 | 109 | 1.39 | 0.95–2.02 |
| *P* for trend | | | 0.073 | |
| Age at leaving school (years) | | | | |
| ≤ 14 | 190 | 190 | 1.00 | |
| 15–16 | 79 | 74 | 1.08 | 0.72–1.63 |
| 17 + | 59 | 64 | 0.92 | 0.60–1.41 |
| *P* for trend | | | 0.751 | |
| Social class | | | | |
| I | 35 | 32 | 1.00 | |
| II | 89 | 113 | 0.68 | 0.38–1.22 |
| III non-manual | 24 | 38 | 0.59 | 0.29–1.21 |
| III manual | 121 | 87 | 1.31 | 0.74–2.33 |
| IV | 44 | 43 | 0.95 | 0.48–1.88 |
| V | 15 | 15 | 0.89 | 0.37–2.14 |
| I, II, III non-manual | 148 | 183 | 1.00 | |
| III manual, IV, V | 180 | 145 | 1.63 | 1.17–2.27 |
| Cigarette smoking | | | | |
| Never | 79 | 82 | 1.00 | |
| Previous | 193 | 191 | 1.05 | 0.72–1.55 |
| Current | 56 | 55 | 1.06 | 0.66–1.70 |
| Father had prostate cancer | | | | |
| No | 321 | 324 | 1.00 | |
| Yes | 7 | 4 | 1.75 | 0.51–5.98 |
| Brother had prostate cancer | | | | |
| No | 319 | 325 | 1.00 | |
| Yes | 9 | 3 | 3.00 | 0.81–11.08 |

© Cancer Research Campaign 1997

**Table 3** Geometric mean daily nutrient intakes in cases and controls (not including supplements)

| Nutrient | Cases ($n$ = 328) | Controls ($n$ = 328) | $P$[a] |
|---|---|---|---|
| Energy (mJ) | 11.1 | 11.1 | 0.729 |
| Carbohydrate (g) | 303 | 301 | 0.799 |
| Starch (g) | 149 | 145 | 0.261 |
| Sugar (g) | 146 | 148 | 0.586 |
| Protein (g) | 84.6 | 86.5 | 0.184 |
| Total fat (g) | 115.6 | 115.9 | 0.910 |
| Saturated fatty acids (g) | 46.9 | 47.2 | 0.797 |
| Monounsaturated fatty acids (g) | 39.3 | 39.5 | 0.870 |
| Polyunsaturated fatty acids (g) | 18.4 | 18.5 | 0.865 |
| Cholesterol (mg) | 341 | 351 | 0.297 |
| Alcohol[b] (g) | 8.46 | 8.47 | 0.988 |
| Non-starch polysaccharides (g) | 16.5 | 16.9 | 0.231 |
| Potassium (mg) | 3602 | 3714 | 0.055 |
| Calcium (mg) | 1128 | 1145 | 0.489 |
| Magnesium (mg) | 338 | 346 | 0.172 |
| Iron (mg) | 12.9 | 13.2 | 0.338 |
| Copper (mg) | 1.51 | 1.51 | 0.933 |
| Zinc (mg) | 9.77 | 10.04 | 0.127 |
| Manganese (mg) | 4.48 | 4.49 | 0.938 |
| Selenium (μg) | 64.0 | 61.8 | 0.231 |
| Iodine (μg) | 131 | 136 | 0.078 |
| Retinol (μg) | 1298 | 1324 | 0.684 |
| Carotene (μg) | 2703 | 2842 | 0.151 |
| Lycopene[c] (μg) | 448 | 462 | 0.723 |
| Vitamin D (μg) | 4.30 | 4.37 | 0.723 |
| Vitamin E (mg) | 12.4 | 12.5 | 0.803 |
| Thiamin (mg) | 1.52 | 1.53 | 0.692 |
| Riboflavin (mg) | 2.24 | 2.28 | 0.364 |
| Niacin (mg) | 20.1 | 20.6 | 0.245 |
| Vitamin $B_6$ (mg) | 1.99 | 2.06 | 0.078 |
| Vitamin $B_{12}$ (μg) | 7.36 | 7.63 | 0.312 |
| Folate (μg) | 328 | 331 | 0.542 |
| Pantothenate (mg) | 12.2 | 12.6 | 0.683 |
| Biotin (μg) | 53.7 | 54.2 | 0.624 |
| Vitamin C (mg) | 77.0 | 78.5 | 0.591 |

[a]Paired $t$-test. [b]Geometric mean of (alcohol intake + 1 g). [c]Geometric mean of (lycopene intake + 1 μg).

the European Prospective Investigation into Cancer and Nutrition (EPIC; Riboli, 1992), which was itself based on the questionnaire developed for the US Nurses' study (Willett et al, 1988). The EPIC food frequency questionnaire has been validated among women in Cambridge (Bingham et al, 1994, 1995). This showed that the questionnaire provides a reasonable estimate of usual intake of important nutrients. For example, for total fat and carotene, which were the primary interests of the current study, the correlations between the estimate of nutrient density from the food frequency questionnaire and from 28 days of weighed dietary records were 0.63 and 0.55 respectively (Bingham et al, 1995).

The food frequency questionnaire enquired about usual frequency of consumption of 83 groups of food items during the last 5 years. Average nutrient intakes were estimated using standard portion sizes, largely from the Ministry of Agriculture Fisheries and Food (1993), and nutrient contents from the fifth edition of McCance and Widdowson's *The Composition of Foods* and its supplements (Holland et al, 1991*a*, 1992*a,b*, 1993; Chan et al, 1994). Carotene is given as β-carotene equivalents (β-carotene + 0.5 × (α-carotene + α-cryptoxanthin + β-cryptoxanthin); Holland et al, 1991*a*). Lycopene (μg) was estimated as carotene from tomatoes and tomato products (mg) multiplied by $1.68 \times 10^{-3}$ (values from Holland et al, 1991*b*).

Participants were asked whether they had regularly taken any vitamin pills or other nutritional supplements during the previous 5 years. Supplement use was slightly less frequent among cases than among controls for all supplements except garlic (Table 1). This information was used to estimate total intakes from food plus supplements of retinol, vitamin E, vitamin $B_6$ and vitamin C using the following assumed vitamin contents of supplements: cod liver oil or other fish oil, 800 μg retinol; multivitamins, 800 μg retinol, 10 mg vitamin E, 2 mg vitamin $B_6$, 50 mg vitamin C; evening primrose oil, 10 mg vitamin E; vitamin A, 800 μg retinol; vitamin B complex or Brewer's yeast, 2 mg vitamin $B_6$; vitamin C, 200 mg vitamin C; and vitamin E, 50 mg vitamin E. These contents were chosen to be representative of common supplements on the market in England (Proprietary Association of Great Britain, 1996).

### Statistical methods

Descriptive statistics were calculated using SPSS (SPSS, Chicago, USA). Estimated nutrient intakes were logarithmically transformed to produce approximately normal distributions, and the mean values quoted are geometric means. Relative risks were estimated as odds ratios (and 95% confidence intervals), calculated with EGRET (Statistics and Epidemiological Research Corporation, 1989) using conditional logistic regression methods for individually matched case–control studies (Breslow and Day, 1980). For nutrients, odds ratios were calculated for thirds of the distribution among controls, and tests for trend were for the logarithm of actual nutrient intake; for foods, odds ratios were calculated for four categories of consumption, selected to give the most even distribution among the controls, and tests for trend were for the daily frequency of consumption. To allow for the association of fat intake with energy intake, we calculated the percentages of energy supplied by fat and fatty acids and present the odds ratios in relation to per cent energy from fat adjusted for energy intake (Willett, 1990). We also calculated energy-adjusted fat intakes by the residuals method of Willett and Stampfer (1986); these results were similar to those for the percentage energy method and are not presented.

Two-sided $P$-values are quoted. Tests for trend were calculated using the logarithmically transformed continuous estimates of nutrient intake.

### RESULTS

### Response rates

Of 425 eligible cases identified, 328 were interviewed (77.2%), 33 refused (7.8%), for 28 the consultant or general practitioner advised against contact (6.6%), 34 died before an interview could be arranged (8.0%) and two had moved outside the area (0.5%). A total of 94 cases (28.7%) had radiological or microscopic evidence of local or metastatic spread at the time of diagnosis.

Of the 328 first controls selected, 267 were interviewed (81.4%), 42 refused (12.8%) and for 19 the general practitioner advised against contact (5.8%).

### Characteristics of cases and controls

The mean ages of cases and controls were identical at 68.1 years. The distributions of reported height were similar in cases and controls, but there was some evidence that risk was higher in the

© Cancer Research Campaign 1997

**Table 4** Odds ratios (ORs) and 95% confidence intervals (CIs) in relation to daily nutrient intakes

| | | | Unadjusted | | Adjusted for social class | |
|---|---|---|---|---|---|---|
| **Nutrient and daily intake** | **Cases** | **Controls** | **OR** | **95% CI** | **OR** | **95% CI** |
| Energy (mJ) | | | | | | |
| < 10.2 | 111 | 109 | 1.00 | | 1.00 | |
| 10.2–12.3 | 112 | 110 | 1.00 | 0.69–1.44 | 1.02 | 0.70–1.48 |
| ≥ 12.4 | 105 | 109 | 0.95 | 0.65–1.38 | 0.90 | 0.62–1.32 |
| *P* for trend | | | 0.729 | | 0.563 | |
| Total fat (g) | | | | | | |
| < 103.0 | 111 | 109 | 1.00 | | 1.00 | |
| 103.0–134.1 | 115 | 110 | 1.02 | 0.71–1.47 | 1.00 | 0.69–1.45 |
| ≥ 134.2 | 102 | 109 | 0.92 | 0.62–1.35 | 0.85 | 0.57–1.25 |
| *P* for trend | | | 0.909 | | 0.574 | |
| Saturated fatty acids (g) | | | | | | |
| < 40.8 | 113 | 109 | 1.00 | | 1.00 | |
| 40.8–54.2 | 111 | 110 | 0.97 | 0.67–1.40 | 0.95 | 0.66–1.38 |
| ≥ 54.3 | 104 | 109 | 0.92 | 0.62–1.35 | 0.87 | 0.58–1.28 |
| *P* for trend | | | 0.796 | | 0.481 | |
| Monounsaturated fatty acids (g) | | | | | | |
| < 34.9 | 105 | 109 | 1.00 | | 1.00 | |
| 34.9–45.2 | 115 | 110 | 1.08 | 0.75–1.56 | 1.06 | 0.73–1.55 |
| ≥ 45.3 | 108 | 109 | 1.03 | 0.70–1.53 | 0.96 | 0.64–1.43 |
| *P* for trend | | | 0.870 | | 0.543 | |
| Polyunsaturated fatty acids (g) | | | | | | |
| < 15.1 | 118 | 109 | 1.00 | | 1.00 | |
| 15.1–21.9 | 98 | 110 | 0.82 | 0.56–1.20 | 0.82 | 0.55–1.21 |
| ≥ 22.0 | 112 | 109 | 0.94 | 0.64–1.37 | 0.94 | 0.64–1.38 |
| *P* for trend | | | 0.865 | | 0.765 | |
| Total fat (per cent energy)[a] | | | | | | |
| < 37.0 | 111 | 109 | 1.00 | | 1.00 | |
| 37.0–41.1 | 105 | 110 | 0.94 | 0.64–1.39 | 0.86 | 0.58–1.28 |
| ≥ 41.2 | 112 | 109 | 1.02 | 0.69–1.49 | 0.93 | 0.63–1.38 |
| *P* for trend | | | 0.621 | | 0.958 | |
| Saturated fatty acids (per cent energy)[a] | | | | | | |
| < 14.1 | 112 | 109 | 1.00 | | 1.00 | |
| 14.1–16.8 | 88 | 110 | 0.78 | 0.53–1.17 | 0.76 | 0.51–1.15 |
| ≥ 16.9 | 128 | 109 | 1.19 | 0.80–1.78 | 1.12 | 0.74–1.69 |
| *P* for trend | | | 0.962 | | 0.701 | |
| Monounsaturated fatty acids (per cent energy)[a] | | | | | | |
| < 12.5 | 108 | 109 | 1.00 | | 1.00 | |
| 12.5–13.9 | 111 | 110 | 1.02 | 0.70–1.49 | 0.95 | 0.65–1.40 |
| ≥ 14.0 | 109 | 109 | 1.02 | 0.68–1.53 | 0.94 | 0.62–1.43 |
| *P* for trend | | | 0.667 | | 0.950 | |
| Polyunsaturated fatty acids (per cent energy)[a] | | | | | | |
| < 5.0 | 117 | 109 | 1.00 | | 1.00 | |
| 5.0–7.6 | 105 | 110 | 0.88 | 0.60–1.29 | 0.89 | 0.61–1.31 |
| ≥ 7.7 | 106 | 109 | 0.89 | 0.61–1.32 | 0.91 | 0.62–1.35 |
| *P* for trend | | | 0.827 | | 0.851 | |
| Alcohol (g) | | | | | | |
| < 3.6 | 105 | 109 | 1.00 | | 1.00 | |
| 3.6–16.5 | 118 | 110 | 1.12 | 0.76–1.63 | 1.13 | 0.77–1.66 |
| ≥ 16.6 | 105 | 109 | 1.01 | 0.69–1.47 | 1.04 | 0.71–1.54 |
| *P* for trend | | | 0.988 | | 0.914 | |
| Non-starch polysaccharides (g) | | | | | | |
| < 15.0 | 131 | 109 | 1.00 | | 1.00 | |
| 15.0–19.1 | 85 | 110 | 0.64 | 0.43–0.94 | 0.69 | 0.46–1.02 |
| ≥ 19.2 | 112 | 109 | 0.86 | 0.58–1.27 | 0.96 | 0.64–1.44 |
| *P* for trend | | | 0.230 | | 0.533 | |
| Potassium (g) | | | | | | |
| < 3.46 | 125 | 109 | 1.00 | | 1.00 | |
| 3.46–4.09 | 110 | 110 | 0.87 | 0.59–1.26 | 0.91 | 0.62–1.34 |
| ≥ 4.10 | 93 | 109 | 0.74 | 0.51–1.09 | 0.81 | 0.55–1.20 |
| *P* for trend | | | 0.054 | | 0.164 | |
| Copper (mg) | | | | | | |
| < 1.35 | 119 | 108 | 1.00 | | 1.00 | |
| 1.35–1.72 | 99 | 111 | 0.82 | 0.57–1.18 | 0.80 | 0.55–1.17 |
| ≥ 1.73 | 110 | 109 | 0.91 | 0.62–1.32 | 0.86 | 0.59–1.25 |
| *P* for trend | | | 0.933 | | 0.779 | |

© Cancer Research Campaign 1997

**Table 4** cont.

| | | | Unadjusted | | Adjusted for social class | |
|---|---|---|---|---|---|---|
| **Nutrient and daily intake** | **Cases** | **Controls** | **OR** | **95% CI** | **OR** | **95% CI** |
| Zinc (mg) | | | | | | |
| < 9.15 | 127 | 109 | 1.00 | | 1.00 | |
| 9.15–10.99 | 106 | 110 | 0.80 | 0.54–1.18 | 0.84 | 0.56–1.25 |
| ≥ 11.00 | 95 | 109 | 0.73 | 0.49–1.08 | 0.78 | 0.52–1.17 |
| *P* for trend | | | 0.126 | | 0.229 | |
| Iodine (μg) | | | | | | |
| < 120 | 122 | 109 | 1.00 | | 1.00 | |
| 120–155 | 113 | 110 | 0.90 | 0.62–1.31 | 0.90 | 0.61–1.32 |
| ≥ 156 | 93 | 109 | 0.75 | 0.51–1.11 | 0.77 | 0.52–1.14 |
| *P* for trend | | | 0.077 | | 0.108 | |
| Retinol, food only (μg) | | | | | | |
| < 944 | 115 | 109 | 1.00 | | 1.00 | |
| 944–1851 | 105 | 110 | 0.90 | 0.62–1.32 | 0.88 | 0.61–1.29 |
| ≥ 1852 | 108 | 109 | 0.94 | 0.66–1.35 | 0.90 | 0.62–1.30 |
| *P* for trend | | | 0.683 | | 0.514 | |
| Retinol, including supplements (μg) | | | | | | |
| < 1214. | 111 | 109 | 1.00 | | 1.00 | |
| 1214–2086 | 117 | 110 | 1.04 | 0.72–1.50 | 1.07 | 0.74–1.56 |
| ≥ 2087 | 100 | 109 | 0.91 | 0.64–1.32 | 0.90 | 0.62–1.30 |
| *P* for trend | | | 0.519 | | 0.441 | |
| Carotene (mg)[b] | | | | | | |
| < 2.65 | 137 | 109 | 1.00 | | 1.00 | |
| 2.65–3.47 | 88 | 110 | 0.65 | 0.45–0.94 | 0.69 | 0.47–1.01 |
| ≥ 3.48 | 103 | 109 | 0.76 | 0.53–1.10 | 0.83 | 0.57–1.21 |
| *P* for trend | | | 0.150 | | 0.351 | |
| Lycopene (μg) | | | | | | |
| < 402 | 117 | 109 | 1.00 | | 1.00 | |
| 402–717 | 103 | 110 | 0.89 | 0.62–1.26 | 0.90 | 0.63–1.29 |
| ≥ 718 | 108 | 109 | 0.93 | 0.64–1.35 | 0.99 | 0.68–1.45 |
| *P* for trend | | | 0.727 | | 0.882 | |
| Vitamin $B_6$, food only (mg) | | | | | | |
| < 1.90 | 128 | 109 | 1.00 | | 1.00 | |
| 1.90–2.26 | 101 | 110 | 0.78 | 0.54–1.13 | 0.85 | 0.58–1.24 |
| ≥ 2.27 | 99 | 109 | 0.77 | 0.53–1.12 | 0.86 | 0.59–1.26 |
| *P* for trend | | | 0.077 | | 0.204 | |
| Vitamin $B_6$, including supplements (mg) | | | | | | |
| < 1.93 | 130 | 107 | 1.00 | | 1.00 | |
| 1.93–2.35 | 104 | 111 | 0.77 | 0.54–1.12 | 0.84 | 0.58–1.23 |
| ≥ 2.36 | 94 | 110 | 0.70 | 0.48–1.03 | 0.79 | 0.54–1.18 |
| *P* for trend | | | 0.029 | | 0.122 | |
| Vitamin C, food only (mg) | | | | | | |
| < 66.1 | 114 | 109 | 1.00 | | 1.00 | |
| 66.1–97.3 | 100 | 110 | 0.89 | 0.62–1.26 | 0.99 | 0.69–1.43 |
| ≥ 97.4 | 114 | 109 | 1.01 | 0.69–1.46 | 1.22 | 0.82–1.81 |
| *P* for trend | | | 0.590 | | 0.607 | |
| Vitamin C, including supplements (mg) | | | | | | |
| < 67.1 | 107 | 109 | 1.00 | | 1.00 | |
| 67.1–104.2 | 112 | 110 | 1.04 | 0.72–1.49 | 1.19 | 0.81–1.74 |
| ≥ 104.3 | 109 | 109 | 1.02 | 0.70–1.48 | 1.23 | 0.83–1.84 |
| *P* for trend | | | 0.390 | | 0.818 | |
| Vitamin E, food only (mg) | | | | | | |
| < 9.59 | 116 | 109 | 1.00 | | 1.00 | |
| 9.59–16.33 | 107 | 110 | 0.90 | 0.60–1.35 | 0.94 | 0.62–1.41 |
| ≥ 16.34 | 105 | 109 | 0.90 | 0.61–1.32 | 0.93 | 0.63–1.37 |
| *P* for trend | | | 0.803 | | 0.890 | |
| Vitamin E, including supplements (mg) | | | | | | |
| < 9.94 | 119 | 109 | 1.00 | | 1.00 | |
| 9.94–17.87 | 112 | 109 | 0.93 | 0.64–1.37 | 0.95 | 0.65–1.41 |
| ≥ 17.88 | 97 | 110 | 0.81 | 0.55–1.18 | 0.85 | 0.58–1.25 |
| *P* for trend | | | 0.296 | | 0.413 | |

[a]Adjusted for log energy intake as a continuous variable. [b]β-Carotene equivalents (β-carotene + 0.5 × (α-carotene + α-cryptoxanthin + β-cryptoxanthin)). *P*-values for trend are for the logarithmically transformed continuous estimates of nutrient intake.

© Cancer Research Campaign 1997

**Table 5** Odds ratios (ORs) and 95% confidence intervals (CIs) in relation to frequency of consumption of selected foods

| | | | Unadjusted | | Adjusted for social class | |
|---|---|---|---|---|---|---|
| **Food and frequency of consumption** | **Cases** | **Control** | **OR** | **95% CI** | **OR** | **95% CI** |
| Meat of any type | | | | | | |
| ≤ 4 per week | 64 | 61 | 1.00 | | 1.00 | |
| 5–6 per week | 94 | 84 | 1.06 | 0.67–1.67 | 1.05 | 0.66–1.67 |
| 1 per day | 134 | 133 | 0.95 | 0.60–1.49 | 0.94 | 0.60–1.49 |
| > 1 per day | 36 | 50 | 0.66 | 0.37–1.18 | 0.64 | 0.36–1.14 |
| *P* for trend | | | 0.160 | | 0.133 | |
| Roast or grilled meat of any type | | | | | | |
| < 1 per week | 26 | 35 | 1.00 | | 1.00 | |
| 1 per week | 78 | 67 | 1.57 | 0.86–2.89 | 1.48 | 0.80–2.74 |
| 2–4 per week | 202 | 202 | 1.35 | 0.77–2.38 | 1.29 | 0.73–2.29 |
| 5 + per week | 22 | 24 | 1.23 | 0.56–2.69 | 1.12 | 0.50–2.49 |
| *P* for trend | | | 0.944 | | 0.807 | |
| Fatty fish[a] | | | | | | |
| Never | 58 | 43 | 1.00 | | 1.00 | |
| < 1 per month | 52 | 53 | 0.72 | 0.41–1.27 | 0.74 | 0.42–1.32 |
| 1–3 per month | 124 | 136 | 0.68 | 0.43–1.08 | 0.70 | 0.44–1.12 |
| ≥ 1 per week | 94 | 96 | 0.72 | 0.44–1.18 | 0.78 | 0.47–1.29 |
| *P* for trend | | | 0.149 | | 0.167 | |
| Cooked vegetables except potatoes | | | | | | |
| ≤ 4 per week | 51 | 45 | 1.00 | | 1.00 | |
| 5–6 per week | 52 | 50 | 0.90 | 0.51–1.57 | 0.87 | 0.49–1.54 |
| 1 per day | 162 | 165 | 0.82 | 0.50–1.34 | 0.86 | 0.52–1.42 |
| > 1 per day | 63 | 68 | 0.68 | 0.33–1.41 | 0.71 | 0.34–1.48 |
| *P* for trend | | | 0.341 | | 0.415 | |
| Carrots | | | | | | |
| ≤ 3 per month | 49 | 43 | 1.00 | | 1.00 | |
| 1 per week | 72 | 69 | 0.90 | 0.53–1.55 | 0.93 | 0.54–1.60 |
| 2–4 per week | 175 | 177 | 0.86 | 0.54–1.38 | 0.92 | 0.57–1.49 |
| ≥ 5 per week | 32 | 39 | 0.70 | 0.37–1.34 | 0.75 | 0.39–1.44 |
| *P* for trend | | | 0.324 | | 0.495 | |
| Dark-green leafy vegetables | | | | | | |
| < 1 per month | 93 | 98 | 1.00 | | 1.00 | |
| 1–3 per month | 71 | 71 | 1.08 | 0.66–1.75 | 1.16 | 0.70–1.89 |
| 1 per week | 77 | 72 | 1.16 | 0.72–1.86 | 1.29 | 0.79–2.11 |
| ≥ 2 per week | 87 | 87 | 1.08 | 0.70–1.65 | 1.24 | 0.79–1.94 |
| *P* for trend | | | 0.770 | | 0.820 | |
| Onions | | | | | | |
| ≤ 3 per month | 75 | 79 | 1.00 | | 1.00 | |
| 1 per week | 72 | 65 | 1.16 | 0.73–1.84 | 1.11 | 0.70–1.77 |
| 2–4 per week | 146 | 142 | 1.09 | 0.72–1.65 | 1.07 | 0.70–1.62 |
| ≥ 5 per week | 35 | 42 | 0.89 | 0.52–1.53 | 0.85 | 0.49–1.48 |
| *P* for trend | | | 0.683 | | 0.654 | |
| Garlic | | | | | | |
| Never | 220 | 192 | 1.00 | | 1.00 | |
| < 1 per month | 31 | 32 | 0.83 | 0.45–1.50 | 0.94 | 0.51–1.73 |
| 1–4 per month | 45 | 56 | 0.70 | 0.45–1.08 | 0.77 | 0.49–1.20 |
| ≥ 2 per week | 32 | 48 | 0.56 | 0.33–0.93 | 0.64 | 0.38–1.09 |
| *P* for trend | | | 0.038 | | 0.129 | |
| Garlic, including supplements | | | | | | |
| Never | 215 | 187 | 1.00 | | 1.00 | |
| < 1 per month | 30 | 31 | 0.84 | 0.45–1.54 | 0.95 | 0.51–1.78 |
| 1–4 per month | 43 | 54 | 0.69 | 0.44–1.08 | 0.75 | 0.48–1.19 |
| ≥ 2 per week | 40 | 56 | 0.60 | 0.37–0.96 | 0.68 | 0.41–1.10 |
| *P* for trend | | | 0.117 | | 0.255 | |
| Raw tomatoes | | | | | | |
| ≤ 3 per month | 75 | 78 | 1.00 | | 1.00 | |
| 1 per week | 71 | 63 | 1.18 | 0.74–1.90 | 1.27 | 0.78–2.05 |
| 2–4 per week | 148 | 149 | 1.04 | 0.70–1.53 | 1.12 | 0.76–1.67 |
| ≥ 5 per week | 34 | 38 | 0.93 | 0.53–1.64 | 1.06 | 0.55–1.62 |
| *P* for trend | | | 0.581 | | 0.883 | |
| Cooked tomatoes | | | | | | |
| < 1 per month | 99 | 91 | 1.00 | | 1.00 | |
| 1–3 per month | 55 | 66 | 0.77 | 0.49–1.21 | 0.77 | 0.49–1.22 |
| 1 per week | 101 | 95 | 0.98 | 0.66–1.45 | 0.99 | 0.67–1.47 |
| ≥ 2 per week | 73 | 76 | 0.88 | 0.57–1.36 | 0.92 | 0.59–1.42 |
| *P* for trend | | | 0.468 | | 0.636 | |

© Cancer Research Campaign 1997

**Table 5** cont.

| Food and frequency of consumption | Cases | Control | Unadjusted OR | Unadjusted 95% CI | Adjusted for social class OR | Adjusted for social class 95% CI |
|---|---|---|---|---|---|---|
| Raw green salad | | | | | | |
| ≤ 3 per month | 92 | 102 | 1.00 | | 1.00 | |
| 1 per week | 91 | 83 | 1.20 | 0.80–1.79 | 1.21 | 0.80–1.81 |
| 2–4 per week | 115 | 100 | 1.26 | 0.86–1.84 | 1.35 | 0.91–1.99 |
| 5–6 per week | 30 | 43 | 0.75 | 0.43–1.31 | 0.87 | 0.49–1.56 |
| *P* for trend | | | 0.670 | | 0.817 | |
| Baked beans | | | | | | |
| < 1 per month | 92 | 76 | 1.00 | | 1.00 | |
| 1–3 per month | 91 | 109 | 0.66 | 0.43–1.02 | 0.66 | 0.43–1.02 |
| 1 per week | 103 | 84 | 1.02 | 0.66–1.58 | 0.98 | 0.63–1.52 |
| ≥ 2 per week | 42 | 59 | 0.57 | 0.34–0.95 | 0.52 | 0.31–0.88 |
| *P* for trend | | | 0.130 | | 0.075 | |
| Garden peas | | | | | | |
| ≤ 3 per month | 63 | 62 | 1.00 | | 1.00 | |
| 1 per week | 107 | 93 | 1.17 | 0.74–1.87 | 1.14 | 0.71–1.83 |
| 2–4 per week | 152 | 155 | 0.99 | 0.65–1.52 | 0.98 | 0.64–1.51 |
| ≥ 5 per week | 6 | 18 | 0.35 | 0.13–0.91 | 0.35 | 0.13–0.94 |
| *P* for trend | | | 0.066 | | 0.081 | |
| Green beans, broad beans, runner beans | | | | | | |
| < 1 per month | 58 | 42 | 1.00 | | 1.00 | |
| 1–3 per month | 68 | 75 | 0.60 | 0.35–1.06 | 0.62 | 0.35–1.09 |
| 1 per week | 96 | 99 | 0.65 | 0.38–1.10 | 0.68 | 0.40–1.16 |
| ≥ 2 per week | 106 | 112 | 0.62 | 0.36–1.07 | 0.66 | 0.38–1.13 |
| *P* for trend | | | 0.362 | | 0.469 | |
| Dried lentils, beans, peas | | | | | | |
| Never | 202 | 200 | 1.00 | | 1.00 | |
| < 1 per month | 31 | 36 | 0.85 | 0.50–1.45 | 0.87 | 0.51–1.50 |
| 1–3 per month | 58 | 62 | 0.92 | 0.60–1.40 | 0.89 | 0.58–1.37 |
| ≥ 1 per week | 37 | 30 | 1.21 | 0.72–2.04 | 1.38 | 0.81–2.36 |
| *P* for trend | | | 0.319 | | 0.144 | |
| Total legumes[b] | | | | | | |
| < 3 per week | 100 | 90 | 1.00 | | 1.00 | |
| 3–4 per week | 92 | 85 | 0.96 | 0.64–1.45 | 0.96 | 0.64–1.46 |
| 5–6 per week | 77 | 89 | 0.76 | 0.49–1.18 | 0.78 | 0.50–1.21 |
| ≥ 1 per day | 59 | 64 | 0.82 | 0.53–1.29 | 0.83 | 0.53–1.30 |
| *P* for trend | | | 0.068 | | 0.093 | |
| Citrus fruit | | | | | | |
| Never | 74 | 81 | 1.00 | | 1.00 | |
| ≤ 1 per week | 118 | 128 | 1.03 | 0.68–1.55 | 1.11 | 0.73–1.69 |
| 2–4 per week | 87 | 75 | 1.29 | 0.82–2.03 | 1.41 | 0.89–2.24 |
| ≥ 5 per week | 49 | 44 | 1.26 | 0.73–2.16 | 1.45 | 0.83–2.52 |
| *P* for trend | | | 0.175 | | 0.091 | |
| Non-citrus fruit | | | | | | |
| < 1 per month | 39 | 37 | 1.00 | | 1.00 | |
| 1–4 per month | 59 | 53 | 1.06 | 0.60–1.88 | 1.18 | 0.65–2.11 |
| 2–4 per week | 90 | 84 | 1.01 | 0.58–1.76 | 1.15 | 0.65–2.02 |
| ≥ 5 per week | 140 | 154 | 0.85 | 0.51–1.42 | 0.99 | 0.58–1.68 |
| *P* for trend | | | 0.307 | | 0.539 | |
| Tea (cups per day) | | | | | | |
| < 3 | 66 | 65 | 1.00 | | 1.00 | |
| 3–4 | 97 | 122 | 0.77 | 0.48–1.22 | 0.73 | 0.46–1.18 |
| 5–6 | 101 | 81 | 1.23 | 0.76–1.97 | 1.15 | 0.71–1.87 |
| ≥ 7 | 64 | 60 | 1.05 | 0.62–1.78 | 0.94 | 1.15–2.26 |
| *P* for trend | | | 0.253 | | 0.522 | |
| Coffee (cups per day) | | | | | | |
| 0 | 109 | 109 | 1.00 | | 1.00 | |
| 1 | 72 | 81 | 0.88 | 0.58–1.34 | 0.92 | 0.60–1.42 |
| 2 | 72 | 52 | 1.34 | 0.86–2.10 | 1.41 | 0.89–2.21 |
| ≥ 3 | 75 | 86 | 0.85 | 0.54–1.35 | 0.94 | 0.59–1.51 |
| *P* for trend | | | 0.750 | | 0.950 | |

[a]Question referred to 'oily fish, fresh or canned, e.g. mackerel, kippers, tuna, salmon, sardines, herring'. [b]Sum of baked beans, garden peas, green beans, broad beans, runner beans, dried lentils, beans and peas. *P* values for trend are for daily frequency as a continuous variable.

© Cancer Research Campaign 1997

middle and top thirds of body mass index than in the lowest third, at both ages 25 and 45 (Table 2).

Age at leaving school was not associated with risk, but risk did vary significantly with social class. The pattern of variation in risk was irregular when social class was categorized in six groups, perhaps because of small numbers in some groups. Social class was therefore also categorized into two groups, non-manual and manual, and the odds ratio for the latter was 1.63 (95% CI 1.17–2.27). Restriction of the analysis to the 267 matched pairs in which the first control participated increased this estimate (odds ratio 1.86; 1.28–2.70). Social class was strongly related to the intake of many nutrients (data not shown), therefore results for nutrients and foods are presented before and after adjustment for social class (non-manual vs manual).

Risk was not significantly associated with cigarette smoking history. More cases than controls reported that their father or a brother had had prostate cancer (odds ratios 1.75; 0.51–5.98 and 3.00; 0.81–11.08 respectively). These results were not substantially altered by adjusting for social class (results not shown).

### Nutrient intakes

Table 3 shows geometric mean intakes of 35 nutrients in cases and controls. None of the differences was statistically significant. The previous hypotheses related to total fat, saturated fat, carotene and lycopene. Geometric mean intakes of these nutrients were lower in cases than in controls: 0.3%, 0.6%, 4.9% ($P = 0.151$) and 3.0% for total fat, saturated fat, carotene and lycopene respectively. Differences were close to significant ($0.05 < P < 0.1$) for potassium, iodine and vitamin $B_6$, for which geometric mean intakes were 3.0%, 3.7% and 3.4% lower respectively in cases than in controls.

Table 4 shows the odds ratios for increasing consumption of 17 nutrients, selected because of a previous hypothesis (total fat, saturated fatty acids, carotene, lycopene), because they are related to fat intake (energy, monounsaturated fatty acids, polyunsaturated fatty acids), because we thought at the time of statistical analysis that they might be associated with prostate cancer (alcohol, non-starch polysaccharides, copper, zinc, retinol, vitamin C, vitamin E), or because the preliminary analyses summarized in Table 3 showed that the difference between cases and controls was close to significant (potassium, iodine, vitamin $B_6$). There was no evidence for an increase in risk with increasing consumption of total fat or of saturated, monounsaturated or polyunsaturated fatty acids, using either actual fat intake or the percentage of energy supplied by fat. There was some evidence that risk was lower in the middle and top thirds of carotene intake than in the bottom third, but the relationship was not linear and the odds ratios increased towards unity after adjusting for social class. There was no suggestion that risk was associated with lycopene. Of the other nutrients examined, the only significant trend was for vitamin $B_6$ including supplements; this association was reduced and no longer statistically significant after adjusting for social class. The only other nutrients for which odds ratios were less than 0.8 in the top third of intake were potassium (unadjusted for social class), zinc and iodine.

### Intake of selected foods

Table 5 shows the association of risk with frequency of consumption of 18 foods and two drinks. These were selected because they were related to the nutrient hypotheses (meat, cooked vegetables, carrots, dark-green leafy vegetables, raw tomatoes, cooked tomatoes, raw salad) or because previous research had suggested that they might be associated with prostate cancer (roast meat, fatty fish, onions, garlic, baked beans, garden peas, green beans, dried lentils, total legumes, citrus fruit, non-citrus fruit, tea, coffee). The only statistically significant results were for garlic (food only or food plus supplements), baked beans and garden peas. After adjusting for social class the association with garlic was reduced and was not statistically significant, but the reductions in risk for frequent consumption of baked beans and garden peas remained significant. Odds ratios in the highest frequency category, unadjusted for social class, were also below 0.8 for meat, fatty fish, cooked vegetables, carrots, raw green salad and green beans (including broad beans and runner beans).

The calculations presented in Tables 4 and 5 were repeated, restricting the analysis to the 94 cases (and their matched controls) for whom there was radiological or microscopic evidence of local or metastatic disease spread at the time of diagnosis. None of these results was statistically significant. The odds ratios (95% confidence intervals) in the top thirds of the distribution of intake of per cent energy from fat, per cent energy from saturated fatty acids, carotene, and lycopene were 1.22 (0.63–2.37), 0.86 (0.42–1.74), 1.21 (0.61–2.40) and 1.65 (0.77–3.51) respectively.

### DISCUSSION

Case–control studies of nutritional factors are susceptible to bias because of over-representation of health conscious people among the controls. It is therefore important to achieve a high response rate among controls. In this study the response rate among first controls was 81.4%. This is higher than the response rate in most previous case–control studies of nutrition and prostate cancer, but some potential for bias remains and the results should therefore be interpreted cautiously.

Another concern with epidemiological studies of prostate cancer is that some of the cases diagnosed after transurethral prostatectomy for benign prostatic enlargement have a disease that would never have progressed to clinical prostate cancer. Restricting the analysis to cases with advanced disease may reduce this problem, but may also introduce bias because of the removal of more educated case patients who may present with more localized cancers and who may be more health-conscious in their dietary habits (Whittemore et al, 1995). Whittemore et al (1995) found that restricting their analysis to cases with advanced disease (and to controls with normal serum prostate-specific antigen concentrations) increased the magnitude of the relationship they observed between saturated fat intake and risk, but in their study there was also a significant relationship between saturated fat and risk when all cases and controls were analysed. In our study the results were not materially altered by restricting the analysis to cases with radiological or microscopic evidence of local or metastatic spread of disease at the time of diagnosis.

Height was not associated with risk, but there was some evidence that risk was greater in subjects with a greater body mass index, both at age 25 years and at age 45 years. The results of previous studies have varied somewhat, but in a review of ten studies the men with the highest body mass index had on average a 25% higher risk for prostate cancer than the thinnest men (Key, 1995), and two subsequent prospective studies have also supported this association (Chyou et al, 1994; Grönberg et al, 1996). This small increase in risk in association with a high body mass index might be mediated by a decrease in the plasma concentration of sex hormone-binding globulin associated with obesity (Gann et al, 1996) or might be due to a higher muscle mass indicative of higher androgen levels (Kolonel, 1996).

© Cancer Research Campaign 1997

Age at leaving school was not associated with risk, but risk was on average higher in the manual than the non-manual classes. The standardized mortality ratio for prostate cancer in England and Wales in 1911 was 92% higher in social class I than in social class V, but by 1971 this gradient had reversed and mortality was 26% higher in social class V than in social class I (Logan, 1982). Other studies in other countries have not established a clear socio-economic gradient for this cancer (Nomura and Kolonel, 1991). We decided to present all odds ratios, both unadjusted and adjusted for social class, but it should be borne in mind that a change in an odds ratio towards unity after adjustment for social class could mean that the factor is biologically related to risk and actually explains some of the observed variation in risk with social class.

A history of prostate cancer in fathers and brothers was associated with a two- to threefold increase in the risk for prostate cancer. This is similar to the results of other studies (Nomura and Kolonel, 1991), although the absolute rates of reporting disease in fathers and brothers were low, probably due to some under-reporting plus, for fathers, the lower incidence rates of prostate cancer in England and Wales a generation ago (Whittemore, 1994).

Eight out of ten studies reviewed in 1994 showed some increase in risk with high fat intakes (Key, 1995), and this was also observed in another large recent case–control study (Whittemore et al, 1995). In the current study, risk was not associated with the intake of total fat or of saturated, monounsaturated or polyunsaturated fatty acids. There was also no suggestion of a positive association between total meat intake and risk – indeed, the lowest odds ratio was for men who ate meat most frequently. The results for fat were not altered by adjusting for energy intake by the nutrient density method or by the residuals method of Willett and Stampfer (1986), and energy itself was not associated with risk. However, in our study the average fat intake was high and the range of fat intakes was narrow, with a median intake of 34.3% of energy from fat in the lowest third. Our results do not provide information on the relationship of low fat intakes with the risk for prostate cancer.

Nine studies reviewed in 1994 and a recent report from another prospective study showed, on average, no association between carotene intake and risk (Key, 1995; Daviglus et al, 1996). Our study suggests some reduction in risk in association with higher carotene intakes, but the trend was irregular and not statistically significant. The absence of any protective effect of β-carotene in the Alpha-Tocopherol, Beta Carotene Prevention Study (1994) or in the Physicians' Health Study (this included 1047 incident prostate cancers: Hennekens et al, 1996) suggests that this carotenoid does not reduce the risk for prostate cancer and that any consistent associations observed in observational studies are probably due to other correlated dietary factors. Giovannucci et al (1995) observed a protective association of lycopene from tomatoes. One other study has reported a protective association for tomatoes (Mills et al, 1989), but two other studies have found no association (Schuman et al, 1982; Le Marchand et al, 1991). Our results did not suggest a reduction in risk in association with estimated lycopene intake, but our estimate was crude and took no account of the wide variation in the bioavailability of lycopene between foods. In Britain, tinned baked beans (see below) may be a major effective source of lycopene because of the high bioavailability of lycopene from the tomato sauce.

Hayes et al (1996) recently reported a significant trend of increasing risk with increasing alcohol consumption in a large case–control study. However, there was no evidence for such a trend in our study or in most previous studies (Key, 1995).

The possibility that vitamin E might reduce the risk for prostate cancer was raised by the results of the Alpha-Tocopherol, Beta Carotene Prevention Study (1994), which reported 34% fewer cases of prostate cancer in the 50 mg of alpha-tocopherol per day arm of the trial. Our results for estimated vitamin E intake do not support this hypothesis, but median vitamin E intake (food and supplements) in the top third was estimated as only 23.9 mg per day. It is nevertheless intriguing that five controls but only one case reported taking vitamin E supplements.

We observed a reduction in risk with increasing consumption of vitamin $B_6$. This association was reduced by adjusting for social class and was not a previous hypothesis, but deficiency of this vitamin results in 'increased and prolonged nuclear uptake of steroid hormones and enhanced end-organ sensitivity to hormone action ... which may be relevant in the aetiology of cancer of the prostate' (Bender, 1994). This hypothesis deserves further investigation.

Among the foods and food groups we examined there were significant reductions in risk associated with garlic, baked beans and garden peas. The association with garlic was partly explained by social class, but the possible anti-carcinogenic effects of garlic (Steinmetz and Potter, 1991; Dorant et al, 1993) should be examined further in relation to prostate cancer. The associations with baked beans and peas are intriguing because the strongest dietary association with prostate cancer reported by Mills et al (1989) was for beans, lentils and peas, and because these foods have constituents which have been hypothesized to reduce cancer risk (Troll and Wiesner, 1983; Steinmetz and Potter, 1991). Another recent study in England produced results similar to ours, with a crude odds ratio of 0.63 for men who reported eating peas or beans more than once a week in comparison with men who ate these foods less often (Ewings and Bowie, 1996).

Tea and coffee consumption were not associated with risk. Green tea contains chemicals which can inhibit 5α-reductase (Liao and Hiipakka, 1995), and one previous study has reported a protective association for (black) tea among Japanese men in Hawaii (Heilbrun et al, 1986), but no evidence for this was found in a previous study in Britain, where tea consumption is much higher (Kinlen et al, 1988).

In conclusion, the role of nutrition in the aetiology of prostate cancer remains unclear. This study did not support the hypothesized role of fat, and is equivocal in relation to carotene. We think that the possible effects of vitamin $B_6$, garlic, and beans and peas should be examined further.

### ACKNOWLEDGEMENTS

We thank Rosemary Brett, Elizabeth Hilton and Bette Ward for identifying and interviewing the patients and controls; the urologists, general practitioners, radiologists, pathologists, cancer registry staff and other medical staff who helped in identifying, contacting and interviewing patients and controls; and all the men who agreed to participate in this study. This study was supported by the Imperial Cancer Research Fund.

### REFERENCES

Alpha-Tocopherol, Beta Carotene Cancer Prevention Study Group (1994) The effect of vitamin E and beta carotene on the incidence of lung cancer and other cancers in male smokers. *N Engl J Med* **330**: 1029–1035

Armstrong B and Doll R (1975) Environmental factors and cancer incidence and mortality in different countries, with special reference to dietary practices. *Int J Cancer* **15**: 617–631

© Cancer Research Campaign 1997

Bender DA (1994) Novel functions of vitamin $B_6$. *Proc Nutr Soc* **53**: 625–630

Bingham SA, Gill C, Welch A, Day K, Cassidy A, Khaw KT, Sneyd MJ, Key TJA, Roe L and Day NE (1994) Comparison of dietary assessment methods in nutritional epidemiology: weighed records *v.* 24 h recalls, food-frequency questionnaires and estimated-diet records. *Br J Nutr* **72**: 619–643

Bingham SA, Plummer M and Day NE (1995) Energy-adjusted nutrient intakes. *Br J Nutr* **74**: 141–143

Breslow NE and Day NE (1980) *Statistical Methods in Cancer Research.* Vol. 1. *The Analysis of Case-Control Studies.* IARC: Lyon

Chan W, Brown J and Buss DH (1994) *Miscellaneous Foods. Fourth supplement to McCance and Widdowson's The Composition of Foods*, 5th edn. Royal Society of Chemistry: Cambridge

Chyou P-H, Nomura AMY and Stemmermann GN (1994) A prospective study of weight, body mass index and other anthropometric measurements in relation to site-specific cancers. *Int J Cancer* **57**: 313–317

Daviglus ML, Dyer AR, Persky V, Chavez N, Drum M, Goldberg J, Liu K, Morris DK, Shekelle RB and Stamler J (1996) Dietary beta-carotene, vitamin-C and risk of prostate-cancer – results from the Western Electric study. *Epidemiology* **7**: 472–477

Dorant E, van den Brandt PA, Goldbohm RA, Hermus RJJ and Sturmans F (1993) Garlic and its significance for the prevention of cancer in humans: a critical view. *Br J Cancer* **67**: 424–429

Ewings P and Bowie C (1996) A case–control study of cancer of the prostate in Somerset and east Devon. *Br J Cancer* **74**: 661–666

Gann PH, Hennekens CH, Ma J, Longcope C and Stampfer MJ (1996) Prospective study of sex hormone levels and risk of prostate cancer. *J Natl Cancer Inst* **88**: 1118–1126

Giovannucci E, Ascherio A, Rimm EB, Stampfer MJ, Colditz GA and Willett WC (1995) Intake of carotenoids and retinol in relation to risk of prostate cancer. *J Natl Cancer Inst* **87**: 1767–1776

Grönberg H, Damber L and Damber J-E (1996) Total food consumption and body mass index in relation to prostate cancer risk: a case–control study in Sweden with prospectively collected exposure data. *J Urol* **155**: 969–974

Hayes RB, Brown LM, Schoenberg JB, Greenberg RS, Silverman DT, Schwartz AG, Swanson GM, Benichou J, Liff JM, Hoover RN and Pottern LM (1996) Alcohol use and prostate cancer risk in US blacks and whites. *Am J Epidemiol* **143**: 692–697

Heilbrun LK, Nomura A and Stemmermann GN (1986) Black tea consumption and cancer risk: a prospective study. *Br J Cancer* **54**: 677–683

Hennekens CH, Buring JE, Manson JE, Stampfer M, Rosner B, Cook NR, Belanger C, LaMotte F, Gaziano JM, Ridker PM, Willett W and Peto R (1996) Lack of effect of long-term supplementation with beta carotene on the incidence of malignant neoplams and cardiovascular disease. *N Engl J Med* **334**: 1145–1149

Holland B, Welch AA, Unwin ID, Buss DH, Paul AA and Southgate DAT (1991*a*) *McCance and Widdowson's The Composition of Foods*, 5th edn. Royal Society of Chemistry: Cambridge

Holland B, Unwin ID and Buss DH (1991*b*) *Vegetables, Herbs and Spices. Fifth supplement to McCance and Widdowson's The Composition of Foods.* Royal Society of Chemistry: Cambridge

Holland B, Unwin ID and Buss DH (1992*a*) *Fruit and Nuts. First supplement to McCance and Widdowson's The Composition of Foods*, 5th edn. Royal Society of Chemistry: Cambridge

Holland B, Welch AA and Buss DH (1992*b*) *Vegetable Dishes. Second supplement to McCance and Widdowson's The Composition of Foods*, 5th edn. Royal Society of Chemistry: Cambridge

Holland B, Brown J and Buss DH (1993) *Fish and Fish Products. Third supplement to McCance and Widdowson's The Composition of Foods*, 5th edn. Royal Society of Chemistry: Cambridge

Key TJA (1995) Risk factors for prostate cancer. *Cancer Surveys* **23**: 63–77

Kinlen LJ, Willows AN, Goldblatt P and Yudkin J (1988) Tea consumption and cancer. *Br J Cancer* **58**: 397–401

Kolonel LN (1996) Nutrition and prostate cancer. *Cancer Causes Control* **7**: 83–94

Le Marchand L, Hankin JH, Kolonel LN and Wilkens LR (1991) Vegetable and fruit consumption in relation to prostate cancer risk in Hawaii: a reevaluation of the effect of dietary beta-carotene. *Am J Epidemiol* **133**: 215–219

Liao S and Hiipakka RA (1995) Selective inhibition of steroid 5α-reductase isozymes by tea epicatechin-3-gallate and epigallocatechin-3-gallate. *Biochem Biophys Res Commun* **214**: 833–838

Logan WPD (1982) Cancer mortality by occupation and social class 1851–1971. *IARC Scientific Publications* No 36. HMSO: London

Mills PK, Beeson WL, Philips RL and Fraser GE (1989) Cohort study of diet, lifestyle, and prostate cancer in Adventist men. *Cancer* **64**: 599–604

Ministry of Agriculture Fisheries and Food (1993) *Food Portion Sizes,* 2nd edn. London: HMSO

Nomura AMY and Kolonel LN (1991) Prostate cancer: a current perspective. *Am J Epidemiol* **13**: 200–227

Peto R, Doll R, Buckley JD and Sporn MB (1981) Can dietary beta-carotene materially reduce human cancer rates? *Nature* **290**: 201–208

Proprietary Association of Great Britain (1996) OTC Directory 96/97. Communications International Group

Riboli E (1992) Nutrition and cancer: Background and rationale of the European Prospective Investigation into Cancer and Nutrition (EPIC). *Ann Oncol* **3**: 783–791

Schuman LM, Mandel JS, Radke A, Seal U and Halber F (1982) Some selected features of the epidemiology of prostatic cancer: Minneapolis-St. Paul, Minnesota case-control study, 1976–1979. In *Trends in Cancer Incidence*, Magnus K (ed), pp. 345–354. Hemisphere Publishing Corporation: Washington

Statistics and Epidemiological Research Corporation (1989) *EGRET User Manual.* Seattle, WA, USA

Steinmetz KA and Potter JD (1991). Vegetables, fruit and cancer. II. Mechanisms. *Cancer Causes Control* **2**: 427–442

Troll W and Wiesner R (1983) Protease inhibitors: possible anticarcinogens in edible seeds. *Prostate* **4**: 345–349

Whittemore AS (1994) Prostate cancer. *Cancer Surveys* **19/20**: 309–322

Whittemore AS, Kolonel LN, Wu AH, John EM, Gallagher RP, Howe GR, Burch JD, Hankin J, Dreon DM, West DW, Teh C-Z and Paffenbarger RS (1995) Prostate cancer in relation to diet, physical activity, and body size in blacks, whites and Asians in the United States and Canada. *J Natl Cancer Inst* **87**: 652–661

Willett W (1990) Implications of total energy intake for epidemiological analyses. In *Nutritional Epidemiology*, pp. 245–271. Oxford University Press: Oxford

Willett W and Stampfer MJ (1986) Total energy intake: implications for epidemiologic analyses. *Am J Epidemiol* **124**: 17–27

Willett WC, Sampson L, Browne ML, Stampfer MJ, Rosner B, Hennekens CH and Speizer FE (1988) The use of a self-administered questionnaire to assess diet four years in the past. *Am J Epidemiol* **127**: 188–199

© Cancer Research Campaign 1997